# Lycopene: Recent Developments in Stabilization Through Encapsulation

**DOI:** 10.3390/antiox15101285

**Published:** 2026-10-03

**Authors:** Gabriela Râpeanu, Daniela Russo, Marija Banožić, Nicoleta Stănciuc

**Affiliations:** 1 Faculty of Food Science and Engineering, Dunarea de Jos University of Galati, 47, Domneasca Street, 800008 Galati, Romania; gabriela.rapeanu@ugal.ro; 2 CNR-Istituto Officina dei Materiali, c/o Institut Laue Langevin, 71, Avenue des Martyrs CS40220, 38043 Grenoble, France; russo@ill.fr; 3 Faculty of Agriculture and Food Technology, University of Mostar, Biskupa Čule, 88000 Mostar, Bosnia and Herzegovina; marina.bunozic@aptf.sum.ba

**Keywords:** lycopene, encapsulation, stabilization, bioavailability

## Abstract

Due to its unique chemical structure, lycopene may induce multiple health benefits as a natural antioxidant. However, the biological properties of lycopene and, therefore, its wide applications in foods and feed, food supplements, pharmaceutics and cosmetics are limited due to its specific hydrophobic nature and low chemical stability. The specific biological and physicochemical characteristics are due to the presence of 11 conjugated double bonds, which highlights it among the >700 carotenoids discovered, being valued as the most efficient singlet oxygen quencher. However, due to its highly unsaturated hydrocarbon structure, lycopene is highly susceptible to chemical degradation when exposed to high temperatures, oxygen, and light, leading to color fading and the loss of bioactive properties. The stability, bioaccessibility and bioavailability of lycopene may be improved by using encapsulation, such as spray and freeze-drying, emulsions, liposomes, complex coacervation, electrospinning, etc. The main purpose of this study was to highlight the main characteristics of lycopene, the main sources, the principle and key factors of encapsulation, thus covering the most used and effective stabilization methods described in the literature. An important issue covered by this work is related to the bioaccessibility and bioavailability of different isomers of lycopene, with the main purpose of clarifying the mechanism of digestion.

## 1. Introduction

The increasing consumer demand for clean-label, sustainable, and health-oriented foods has led to intensive research in identifying natural pigments as alternatives to synthetic colorants. Therefore, different sources for natural pigments, such as plant and related by-products, algal-and microalgal-derived sources, including anthocyanins, carotenoids, betalains, chlorophylls, curcuminoids, and phycocyanin, are important not only for their color-imparting properties but also for their functional and biological relevance [1,2]. Therefore, besides having a strong visual impact influencing the sensory properties, numerous studies have shown their potential antioxidant, anti-inflammatory, antimicrobial, cardiometabolic, and other health-related properties. These properties are related to the ability to inhibit free radical generation and cellular redox imbalance, associated with several diseases such as cancer, diabetes, cataract, and cardiovascular diseases [3]. Beyond its capacity to directly scavenge free radicals, data from recent literature link lycopene’s antioxidant protection to broader redox signaling—specifically the modulation of Nrf2/ARE-related cytoprotective responses—a factor that helps explain its relevance in several chronic diseases [4]. Benabdelmoumene et al. [5] suggested that both in vitro and in vivo studies demonstrated lycopene’s ability to enhance the endogenous antioxidant defense system by upregulating enzymatic activities of superoxide dismutase, catalase, and glutathione peroxidase. From the perspective of preventing, maintaining or even treating such disorders, the need for daily intake of antioxidants becomes a priority, leading to an unprecedented intensification of efforts to identify, separate and stabilize natural biologically active compounds with antioxidant potential [6]. Moreover, according to Market.us Report [7], it is expected that the carotenoids market (especially referring to astaxanthin, β-carotene, lycopene, lutein, zeaxanthin and canthaxanthin) will yield 3.4 billion USD until the end of 2032, with an estimated CAGR of around 6.3% during the forecasted period (2023–2032).

Moreover, recent technologies go beyond pigment applications, from simple colorant replacement toward more advanced applications, such as in functional food and beverages, fermented dairy and plant products, edible films, intelligent or active packaging and 3D-printed foods [8]. Due to the extended conjugated structures, which support strong color intensity and antioxidant potential, the same structure makes carotenoids, including lycopene, vulnerable to oxidation, photoisomerization, and poor aqueous dispersibility [8]. Considering the structural particularities of each compound, the applications of the colorants impose different tailored-driven preliminary treatment, extraction and stabilization in order to prevent degradation and to improve bioavailability.

Among the >700 carotenoids identified so far, lycopene, due to its structural and chemical particularities, shows a high potential to be used as an effective antioxidant agent in different applications, such as foods and feed, food supplements, drugs, or cosmetics. The structural particularities refer to its long linear chain hydrocarbon (C_40_H_56_) with 13 double bonds (11 conjugated carbon-carbon and 2 non-conjugated). Due to the large number of double bonds in its molecular backbone, lycopene can theoretically assume 2048 geometric configurations, but only a few double bond groups undergo geometric isomerization due to steric hindrance, with the 5*cis*-, 9*cis*-, 13*cis*-, and 15*cis*- isomers predominating, as explained by Caseiro et al. [9].

Lycopene exhibits a singlet-oxygen-quenching ability twice as high as that of β-carotene and ten times higher than that of α-tocopherol [10]. However, lycopene shows gastrointestinal instability and poor water solubility, photosensitivity, and thermal sensitivity [11]. Therefore, the bioavailability of lycopene is low, and its applications in the food industry and related fields are limited. In order to protect lycopene against the effects of storage conditions and to improve its bioaccessibility and bioavailability, encapsulation may lead to highly controllable delivery systems such as emulsions, nanocomplexes, liposomes, and polymeric micelles [12].

The main objective of this work is to identify the main structural characteristics of lycopene that limit its application, provide a short overview of the sources derived from food by-products, and outline the principles and key factors in stabilization. The analyzed literature covered the period 2003–2026 by accessing the Scopus, Web of Science, and PubMed databases, using the main keywords “lycopene” AND/OR “encapsulation”, “microencapsulation AND/OR “nanoencapsulation”, “lycopene bioaccessibility” AND/OR “lycopene bioavailability”, “spray drying”, freeze drying”, “emulsion of lycopene”, “lycopene isomers”, etc. The analyzed literature was selected based on the main scope, emphasizing the main techniques and the differences between the particles, in terms of structure and morphology, influencing the behavior in the gastrointestinal environment and bioaccessibility and bioavailability. Moreover, some additional information is provided on the absorption mechanism of different lycopene isomers.

Moreover, the advantages and disadvantages of different encapsulation techniques are described, emphasizing some combined methods aimed at promoting industrial development and production. Further, different encapsulation methods were described in principle, whereas combined protocols, such as complex coacervation and spray drying and emulsion combined with freeze-drying, were also described, thus covering the most used and effective stabilization methods described in the literature. From the critical analysis of the literature, it was found that these methods are the main technologies applied on a large scale for the encapsulation and stabilization of lycopene.

## 2. The Importance of Reintegration of Bioactive By-Products into Circular Food Systems

FAO [13] estimated that at the global level, agri-food systems are responsible for producing approximately 11 billion tons of food and a multitude of non-food products annually, which generates waste and losses of $750 billion. Food loss and food waste do not pose only social and economic issues but also significant environmental and ethical risks, since between 8.9 and 10.5% of the world’s population faces hunger [14]. Considering the current problems facing society (pandemic, conflicts, malnutrition, climate change, food safety, nutritional security, etc.), a transition from the make-use-waste concept to more circular food systems has become a priority on government agendas, from the perspective of ensuring a more sustainable economy.

The red coloration of various foods is due to the content of lycopene, such as tomatoes, watermelon, papaya, and guava [15]. Different values for lycopene (mg/100 g fresh matter) concentration in foods are given by Benabdelmoumene et al. [16], such as 3.0–7.2 in fresh tomato, 15.0–30.0 in tomato paste, 4.1–5.2 in watermelon, 5.0–7.1 in pink-flesh guava.

To obtain lycopene-enriched extracts, a key factor is the extraction step. In general, extraction methods can be classified as conventional methods, such as solid–liquid extraction using solvents, and advanced methods, such as supercritical fluid extraction. It is important to note that the selection of the extraction method depends on a number of factors, such as the variability of raw material and extraction parameters, such as the type of solvent, solid–liquid ratio, temperature, extraction time, etc. For this reason, to obtain high extraction yields, it is necessary to optimize and validate the extraction conditions, thus allowing scale-up.

Table 1 presents several pieces of information regarding the sources of lycopene extraction from tomato, the main source, together with selected parameters that influenced the extraction yield.

## 3. Basic Principles of Encapsulation

Encapsulation comprises several methods, based on the main principle of trapping one or a mixture of bioactive compounds, named core/active compound(s), within another material, called coating/shell, in order to protect sensitive biofunctional materials from undesired conditions. Therefore, the main objective of encapsulation is to entrap and protect sensitive compounds such as lycopene in a solid or liquid carrier, hence improving their stability and water solubility while increasing the bioaccessibility and bioavailability [22]. It is well known that encapsulation techniques are classified into two main categories, physical and chemical, with the latter including polycondensation methods [23]. Micro- and nanoencapsulation are based on similar technological approaches, with some operational conditions being adapted in order to obtain microparticles or nanoparticles, leading to significant differences in the sizes of particles. Therefore, the size of nanoparticles varies between 1 and 100 nm [24], while the size of microcapsules varies between 0.2~5000 µm or larger. Depending on the applications, different methods are used for lycopene encapsulation, such as spray drying, freeze-drying, coacervation, emulsions, inclusion, ionotropic gelation, etc.

**Figure 1:**
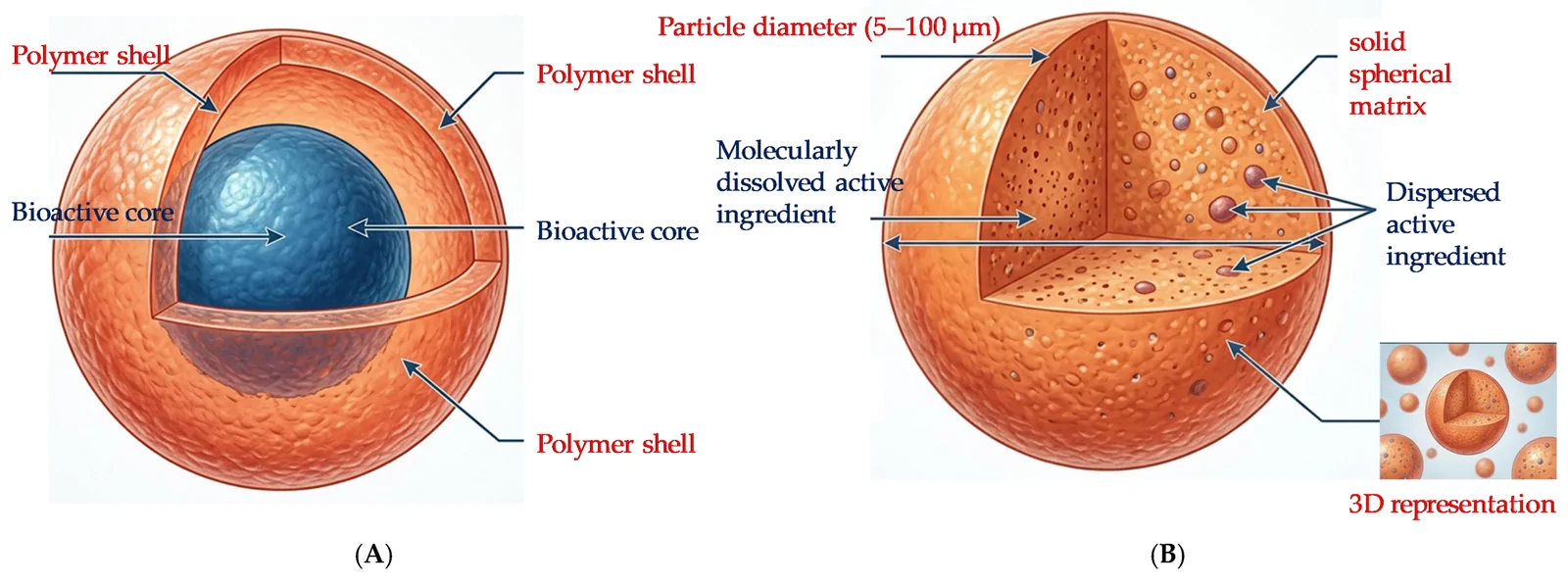
(**A**) Microparticles and (**B**) microsphere.

Regarding the shape and the structure of the capsules, the encapsulation end-products may be divided into microcapsules (reservoir system) and microspheres. Microcapsules are particles that have a centered inner core, containing the bioactive, covered by a polymer layer, while microspheres are defined as a solid spherical matrix in which the active ingredient is molecularly dissolved or heterogeneously dispersed throughout the entire particle volume, rather than isolated in a separate core [25] (Figure 1).

## 4. Short Overview of the Carriers

**Figure 2:**
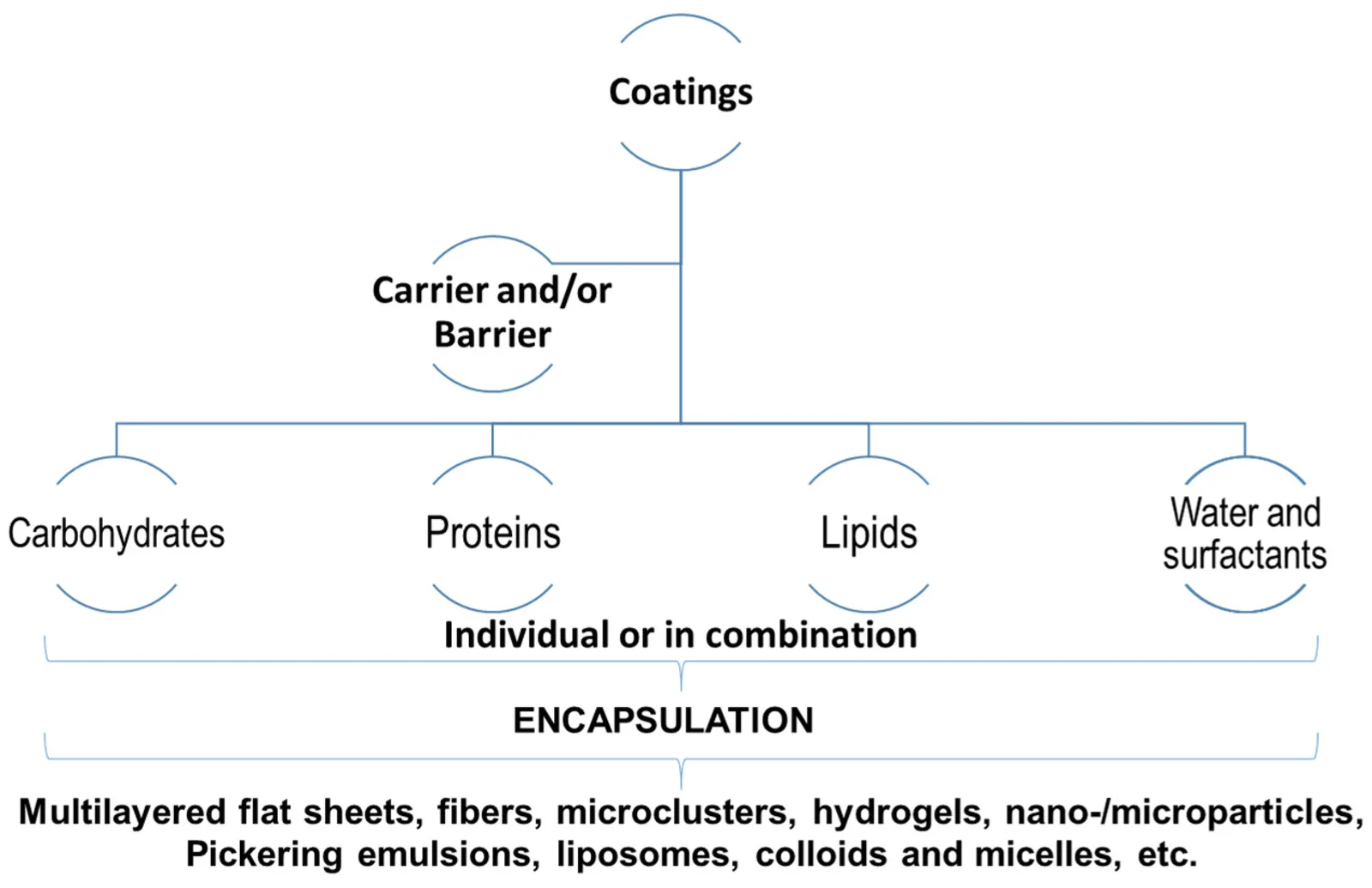
The main coatings used in encapsulation and the main type of particles.

In general, four classes of protective wall materials are used for encapsulation, classified according to their nature, such as lipids, proteins, carbohydrates and gums [26], either alone or in combination (Figure 2). In general, in order to improve de-encapsulation efficiency, the gastrointestinal potential transition and functional properties, different combinations of shells are used [27]. Several factors are involved in the selection of proper carrier materials, such as the properties of the bioactive, the encapsulation method, the nature and chemical structure of the wall materials and the final destination of the end-product [28].

In the literature, different categories of coating materials are studied (Figure 2). A particularly important aspect regarding the properties of coating materials is compatibility and the lack of interaction with the bioactive, while simultaneously ensuring characteristics such as stability, strength, flexibility, impermeability, and non-hygroscopicity.

Four main categories of coating polymers are commonly used. The first category refers to natural polymers, such as polysaccharides (alginate, chitosan, agarose, hyaluronic acid, dextran, etc.) and protein-based polymers (albumin, gelatin). The second category uses synthetic polymers (polycaprolactone, polyethylene glycol, poly(lactic-co-glycolic acid) copolymer, etc.), whereas the third category uses responsive polymers—polymers designed with biologically reactive functional groups, capable of altering their physical and chemical properties in response to exogenous and endogenous factors such as pH, temperature, etc. [28]. Furthermore, in addition to polymers, coating compositions include plasticizers, colorants (in food and pharmaceutical applications), resins, waxes, lipids, and release-rate modifying agents. Some specific examples are cellulose, starch, dextran, agarose, guar gum, alginate, pectin, xanthan, carrageenan, chitosan, whey proteins, soy proteins, casein, triacylglycerols, phospholipids and sterols.

The individual shell highly influences the lycopene-matrix interactions, oxidative protection, gastrointestinal behavior, release mechanism and compatibility with food or nutraceutical applications. For example, the primary bonds in protein, polysaccharides, and lipid-based systems are hydrophobic and hydrogen bonds, physical entrapment and steric exclusion, and hydrophobic dissolution, respectively. The highest oxidative protection is given by the protein–protein interaction, whereas the gastrointestinal behavior is variable, with polysaccharide-based systems assuring the highest protection in gastric juice. Lipid-based systems assure the highest and rapid intestinal release. These properties dictate the applications of encapsulated lycopene in different foods.

Different approaches are used in experimental studies, in terms of the nature and use of carriers, either individually or in combination. Different binary blends for wall material (e.g., gum Arabic, maltodextrin, etc.) using equal quantities of each polymer may be dissolved until complete rehydration of the polymers. The oil-lycopene-rich emulsions may be prepared using different oils (corn, linseed, etc.) as the oil phase. The oils/water emulsions are prepared by adding oil enriched with lycopene drop-by-drop to the biopolymer solution, while homogenizing. Szabo et al. [29] used a ratio of linseed oil enriched with lycopene to biopolymers of 1:4 (*w*/*w*) to obtain the o/w emulsions. It is recommended that, once the oil was added, homogenization continues over an ice bath in order to maintain the system stable for an additional 3 min.

A significant number of studies use whey protein isolates (WPI) and sodium alginate (SA) stock solution as a model for a binary system by dispersing them in double-distilled water, followed by constant stirring until complete hydration. Shu et al. [30] combined the WPI stock solution with the SA solution at a mass ratio of 1:1, whereas the corn oil enriched with lycopene was then added and sheared to obtain an oil-in-water emulsion. In another study, Souza et al. [31] microencapsulated tomato concentrate, enriched in lycopene, in a ratio of concentrate’s solids to encapsulating agent (maltodextrin, whey proteins and the modified starch Capsul^®^) of 1:3 and dried the mixture. The authors suggested that the superior protection of lycopene during storage was obtained when using maltodextrin and Capsul^®^ modified starch, as well as the combination of both.

Another blend used for complex coacervation combined with a freeze-drying encapsulation method involves the use of protein isolate (whey proteins, soy proteins, casein, etc.) and carbohydrate (gum acacia, carboxymethyl cellulose, starch, dextran, agarose, guar gum, alginate, pectin, xanthan, carrageenan, chitosan, etc.) in ultrapure water. Lycopene in sunflower oil was added, followed by homogenization in order to obtain an oil-in-water emulsion. In the emulsion, the carbohydrate solution was added and homogenized. Coacervation was achieved by adjusting the pH of the solutions to 3.75, under continuous stirring. The obtained mixture was cooled in an ice bath under stirring and subsequently stored at 7 °C for 12 h to promote phase separation. The obtained coacervates were freeze-dried under pressure for 48 h [32,33].

## 5. Techniques for Encapsulation and Stabilization of Lycopene

Keeping in mind the abovementioned essential principles on encapsulation, different techniques were used for micro- and nanoencapsulation of lycopene. These techniques include spray drying, freeze-drying, emulsification, liposomal microcapsules, electrospinning, solid lipid nanoparticles, nanostructured lipid carriers, nanoemulsions and nanoliposomes as a single or in combination of micro- and nanoencapsulation methods. Several of these methods will be described, considering technical challenges, limitations, structure and morphology of the particles and a short overview of results from the literature.

### 5.1. Microencapsulation Techniques

#### 5.1.1. Spray Drying Microencapsulation of Lycopene

The use of the spray-drying method allows the conversion of a liquid phase into dry powder through a hot gas stream [34]. The method is cost-effective and simple, but its main drawbacks are the use of high temperatures that could deteriorate heat-sensitive compounds, as well as uncontrolled droplet size, resulting in the formation of irregular particles [35,36].

**Figure 3:**
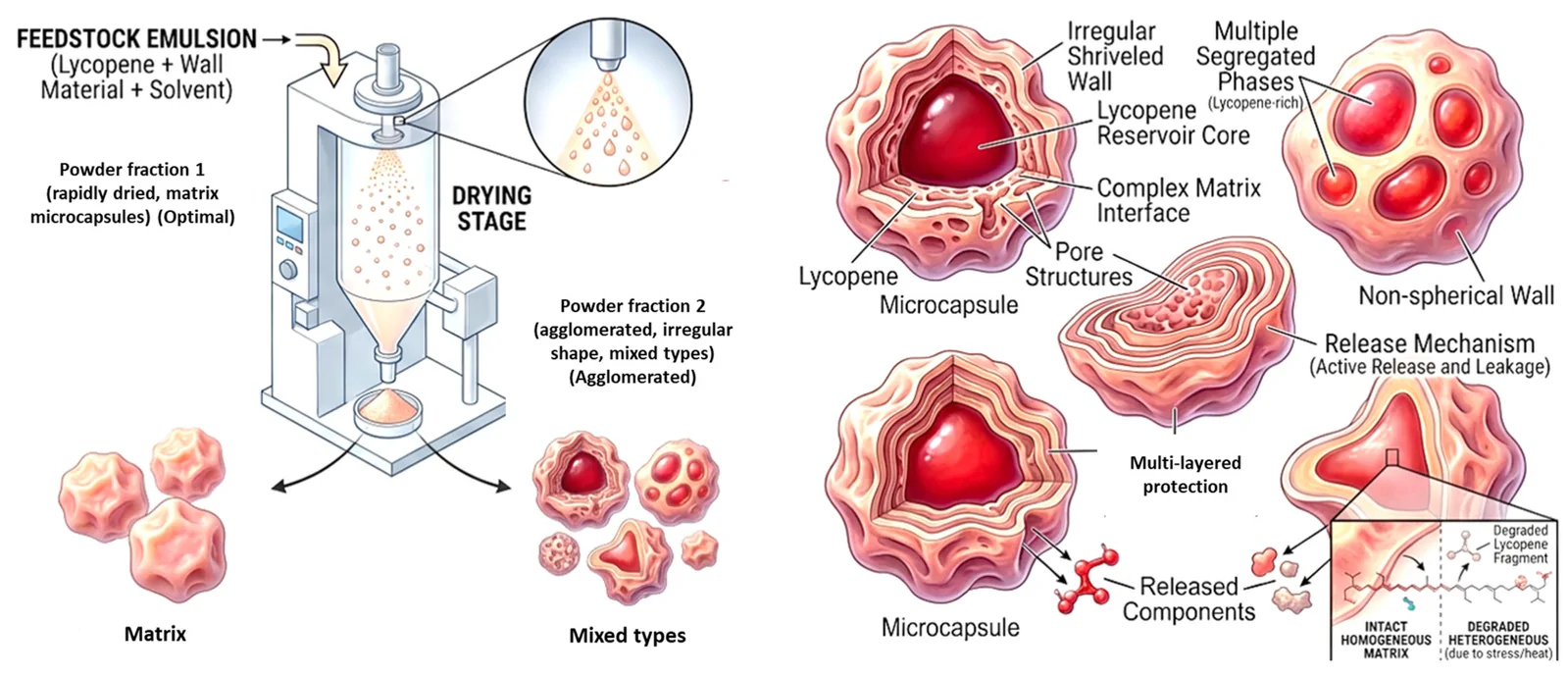
The microencapsulation of lycopene by spray drying and microcapsule analysis.

Figure 3 shows the principles of spray-drying microencapsulation of lycopene, together with the structure of the resulting irregular shapes of the microcapsules.

As can be seen from Figure 3, spray drying produces mostly spherical microparticles, but rapid drying can create dents, shriveled “raisin-like” shapes, or even hollow structures, with dimensions ranging from 10 to 100 micrometers. Souza et al. [31] studied the effect of different combinations of polymers on the physicochemical properties and lycopene stability of a tomato concentrate microencapsulated by spray drying. The powders showed a lycopene content varying from 333 to 494 μg/g. These authors concluded that the best lycopene retention was obtained when using maltodextrin and Capsul^®^ modified starch, as well as the combination of both. These authors suggested that lycopene retention after 28 days of storage at 25 °C indicated high instability in the encapsulated form.

Li et al. [37] used spray drying to microencapsulate lycopene in a combination of polymers, containing maltodextrin and inulin. These authors suggested that encapsulation efficiency varied between 22% and 78%, with a protective effect against degradation during storage. In another study, Corrêa-Filho et al. [38] used inulin and arabic gum as wall materials, at different concentrations of 10 and 20% and drying temperatures of 160 °C and 200 °C, respectively and compared the stability of free lycopene with the microencapsulated one. The microencapsulation allowed obtaining a powder that was more stable over 27 days of storage under light and oxygen conditions. The authors suggested a low encapsulation efficiency and a particle size range of 0.58–26 μm.

Jia et al. [39] used whey protein and isolate-xylo-oligosaccharide conjugates prepared by Maillard reaction as wall material for lycopene microencapsulation using spray drying. The modified carriers allowed obtaining an encapsulation efficiency of 94%. The structure and morphological properties evidenced spherical microcapsules, with a continuous surface and some concavities. The solubility of lycopene was significantly enhanced, whereas the bioaccessibility of lycopene reached a value of 60%. Additionally, these authors suggested that lycopene microcapsules exhibited the same antioxidant capacity as free lycopene; therefore, the use of whey protein glycosylation shows great potential to formulate an efficient and stable delivery system, preserving the antioxidant activity of lycopene.

Hussain et al. [40] reported that drying conditions involved the loading of lycopene-conjugates at a flow rate of 12 mL/min mixing and an inlet and outlet temperatures of 190 °C and 75 °C, correspondingly. The particles showed a complete capacity to dissolve in water and good storage stability related to the water content. The microparticles were sphere-shaped, with a smooth surface and specific irregularities due to drying, with an average diameter of 4.33 ± 1.70 μm. Different storage conditions were tested, showing that the microparticles had the ability to retain 90.79% of lycopene at −20 °C, 67.16% at −4 °C, 54.59% at 25 °C, and 9.64% at 50 °C for 21 days. A slow release of 21.91% lycopene in the gastric phase after 2 h was suggested, whereas after 4 h of digestion, the release of lycopene in the intestinal environment reached 61.20%. The authors explained the sustained release of lycopene due to the structural particularities of the protein-carbohydrate conjugates, which provide a diffusion-layer barrier and offer a steric barrier to the bioactive compound. Additionally, good DPPH antiradical activity was found, suggesting that microencapsulation of lycopene by spray drying imposes a proper design of matrices in order to increase the stability, solubility, and bioavailability, thus extending the potential applications in food and nutraceutical industries.

Zhao et al. [41] studied the impact of basil seed gum concentration and spray drying on the physicochemical, bioaccessibility, and antioxidant properties of encapsulated lycopene emulsion using Central Composite Design-Response Surface Methodology. The authors established the optimal encapsulation condition at an inlet temperature of 141.96 °C and a concentration of basil seed gum of 19.507%. The encapsulated lycopene emulsion showed good colloidal stability, expressed by the average particle size of 147 nm, a polydispersity index of 0.263, and a zeta potential of −21.37 mV. The antioxidant activity was tested during four weeks of storage, with an increase-decrease trend, but overall with stable values. The particles were tested in mayonnaise, significantly influencing the acid value, the anisidine index, the peroxide value, and the TBARS index. As expected, the addition of microencapsulated lycopene emulsion improved color parameters, reducing brightness and whiteness, while masking lycopene’s strong yellow and red hues [42].

#### 5.1.2. Freeze-Drying Microencapsulation of Lycopene

**Figure 4:**
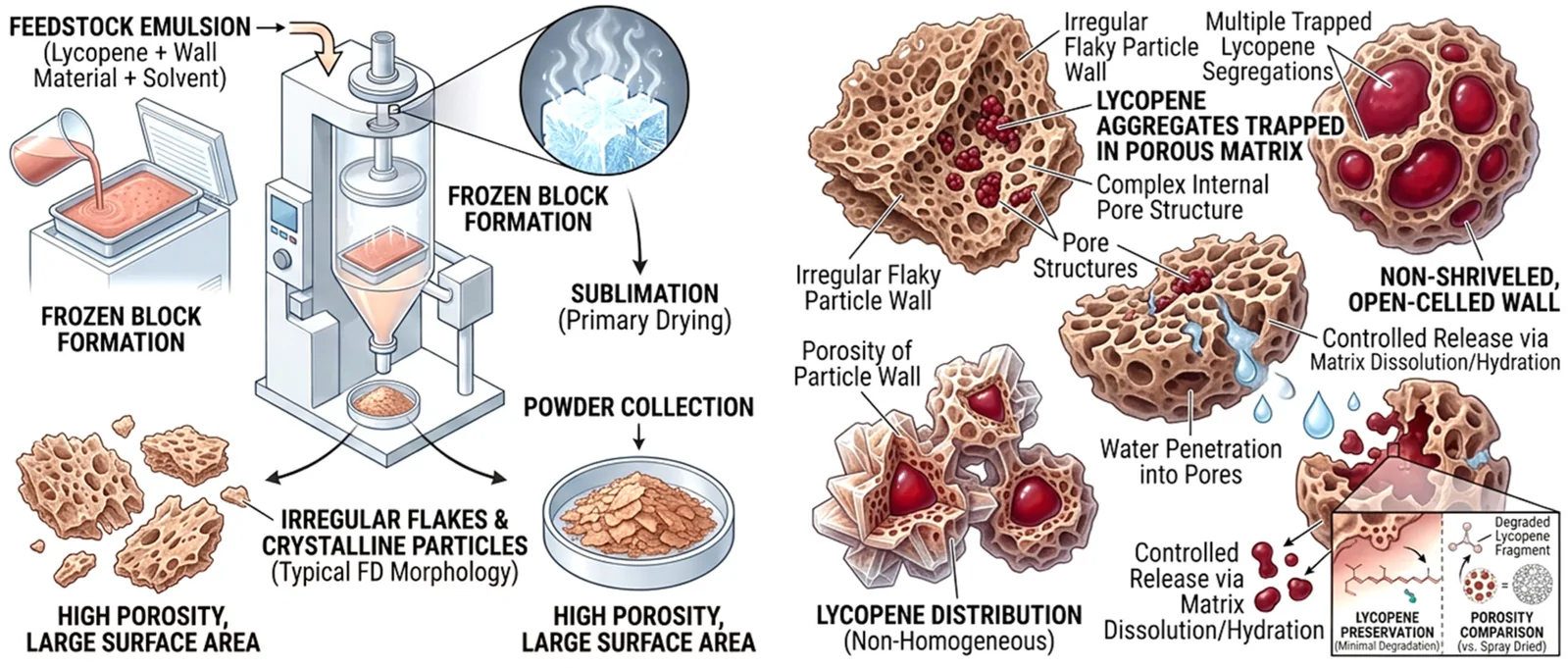
The mechanism of microencapsulation of lycopene by freeze-drying and microcapsule analysis.

A significant number of studies have focused on microencapsulation of lycopene by freeze-drying in order to obtain powders with enhanced properties while minimizing several limiting factors of the process, such as its lengthy processing time, high cost, and the production of dispersed particles. Basically, freeze-drying allows obtaining powders by sublimation of a frozen sample, which is transformed directly into vapor without transitioning through the liquid state [43] (Figure 4).

From Figure 4, one can observe irregular, highly porous, and fragile structures of microcapsules, induced by water sublimation. These microparticles typically have larger dimensions and higher encapsulation efficiencies due to the low-temperature processing, thus preserving the integrity of the bioactive compounds. From the analysis, it can be observed that microparticles show irregular, angular, and flaky rather than smooth spheres, resulting from mechanical grinding or breaking of a continuous frozen matrix. The surface is very porous, wrinkled, and shriveled, due to the growth of ice crystals and subsequent sublimation under vacuum. The size varies from 20 μm to several thousand μm, with a tendency to agglomerate.

As a main advantage, the microencapsulation of bioactive compounds using the freeze-drying method consists of the potential retention of their original nutritional value, texture, and flavor, due to the fact that the technique does not use elevated temperatures. However, as a main disadvantage when compared to alternative technologies, the freeze-drying method involves higher energy consumption and high process costs [35].

Aldana-Heredia et al. [43] used tomato peels and seeds to extract carotenoids, using a microwave-assisted extraction method. The authors suggested a total carotenoid content of 3.19 ± 0.23 mg β-carotene equivalents/g weight, with a high concentration of 5-*cis*-lycopene (1340.1 ± 15.6 μg *all*-*trans*-β-carotene equivalents/g dry weight) and 9-*cis*-lycopene (1062.7 ± 12.8 μg *all*-trans-β-carotene equivalents/g weight). The optimized extract was microencapsulated in two matrices, containing maltodextrin combined with either gum arabic or whey protein isolate as wall materials. The encapsulation efficiency depended on the matrix used, with a value of 68.12 ± 1.42% for maltodextrin and gum arabic and 74.55 ± 1.62% for maltodextrin and whey protein isolate. Bioaccessibility studies for encapsulated extract revealed values of 27.68% ± 0.72 and 25.10% ± 0.04 for the two combinations of polymers. The presence of gum arabic allowed obtaining a higher dissolution time when compared with whey protein, indicating that whey protein will have better solubility in water at room temperature. The morphological analysis showed that in both cases, irregular shapes and several pores on the surface were clearly observed, meaning that the microcapsules were not sealed properly due to the tendency to form crystals during freeze-drying, which are suddenly sublimated, leading to pores on the powder surface [43].

#### 5.1.3. Emulsification

**Figure 5:**
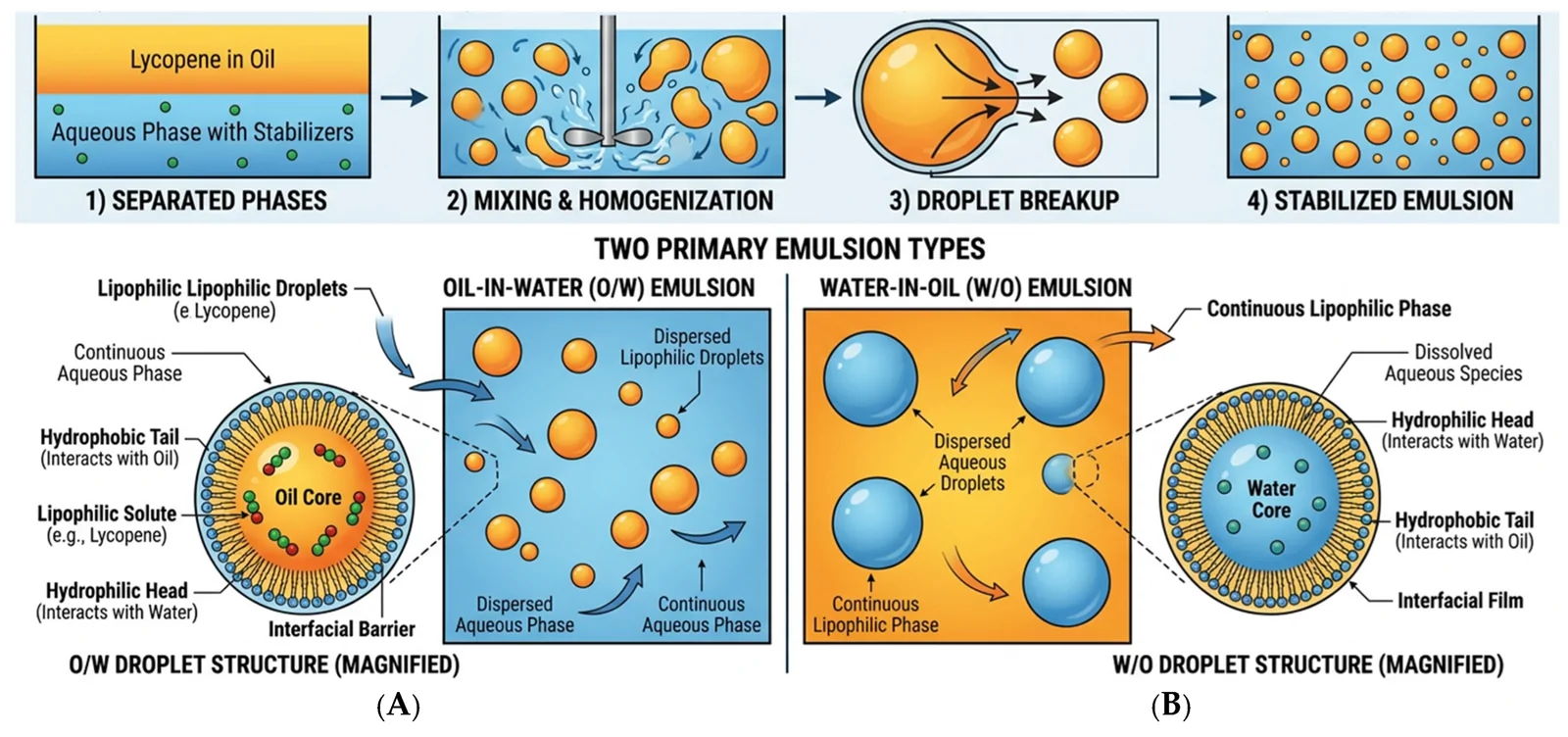
Key factors for droplet formation and stability in emulsification of lycopene (**A**) O/W emulsion and (**B**) W/O emulsion.

Emulsification is a favorable encapsulation method for lipophilic compounds, such as lycopene, due to the presence of a hydrophobic core that can effectively solubilize and transport the targeted encapsulated compound [44]. In general, emulsification is based on droplet formation within a two-phase liquid system where two immiscible solvents are thoroughly homogenized. Two types of emulsions are known, such as oil-in-water (O/W) emulsions and water-in-oil (W/O) emulsions, with the former being the prevalent type employed, as explained by Sereti et al. [24] (Figure 5).

Different studies are related to several key factors in emulsification, mainly related to the oil phase, the type of emulsifiers, antioxidants, metal-chelating agents, homogenization conditions, particle size, etc., from the perspective of improving lycopene stability and related physicochemical and functional properties [22]. In general, microemulsions have the tendency to separate into two phases due to interfacial tension, which necessitates the use of an artificial or natural surfactant [32]. These authors reported that emulsification allows obtaining high microencapsulation efficiency, above 90%, thus improving the bioaccessibility of carotenoids.

Lycopene, a hydrophobic compound, may be easily dissolved in the oil phase, leading to micro- or nanoemulsion systems with improved stability and bioavailability [16]. These authors suggested that the lycopene-loaded emulsions were thermodynamically stable, isotropic, transparent or translucent, homogeneously phase-dispersed and could spontaneously be formed by water, oil, surfactants and cosurfactants with a particle size of 1 to 100 nm. However, it should be kept in mind that due to its chemical stability, the lycopene-loaded emulsion may be easily affected by environmental conditions like temperature or pH changes [44].

Shu et al. [30] used alginate-based emulsion gels to improve the low water solubility and poor bioaccessibility of lycopene. These authors studied the effect of internal and external gelation on the physical properties, water distribution, and lycopene encapsulation properties, using whey protein isolate, sodium alginate and corn oil to prepare an O/W emulsion. In order to induce the external gelation, soluble calcium (CaCl_2_) was added to oil-in-water emulsions containing alginate in the aqueous phase, whereas internal gelation was performed by adding insoluble calcium (CaCO_3_) and D-(+)-gluconic acid δ-lactone to these emulsions. These authors reported different appearance and microstructure of emulsion gels. When compared, the internal emulsion gels presented a higher ability to maintain the specific shape. Moreover, the internal emulsion gels had better formability, smoother surfaces, and were less prone to collapse [45]. The microemulsions formed using the external gelation had rougher surfaces, were less moldable, softer, and had lower water-holding properties than those formed using the internal gelation mechanism.

The bioaccessibility of lycopene depended on the calcium concentration and the method of gelation, with a significant and a non-significant decrease in the case of external and internal gelation, respectively, when increasing the concentration of calcium. The bioaccessibility of lycopene reached ~ 36.5% in the case of internal gelation at 5 mM calcium ions, correlated with faster digestion and release of lycopene from more uniform networks. In another study, Zhao et al. [41] tested two bioactive compounds of hydrophobic (lycopene) and hydrophilic (rutin) nature in two kinds of alginate-based matrices, respectively: calcium alginate gels (solid) or EDTA-calcium alginate sols (fluid). The authors used oleic acid for developing W/O/W emulsions to provide temperature-triggered release, whereas alginate-based matrices were used to provide pH-triggered release. The authors demonstrated a 78% release rate of lycopene in the same phase of the gastrointestinal tract.

Several drawbacks of emulsification were highlighted by Chen et al. [11], related especially to flocculation or aggregation of the emulsion, the complexity of the production, and the use of a high concentration of surfactant, whereas the encapsulation efficiency of lycopene is lower.

#### 5.1.4. Coacervation

**Figure 6:**
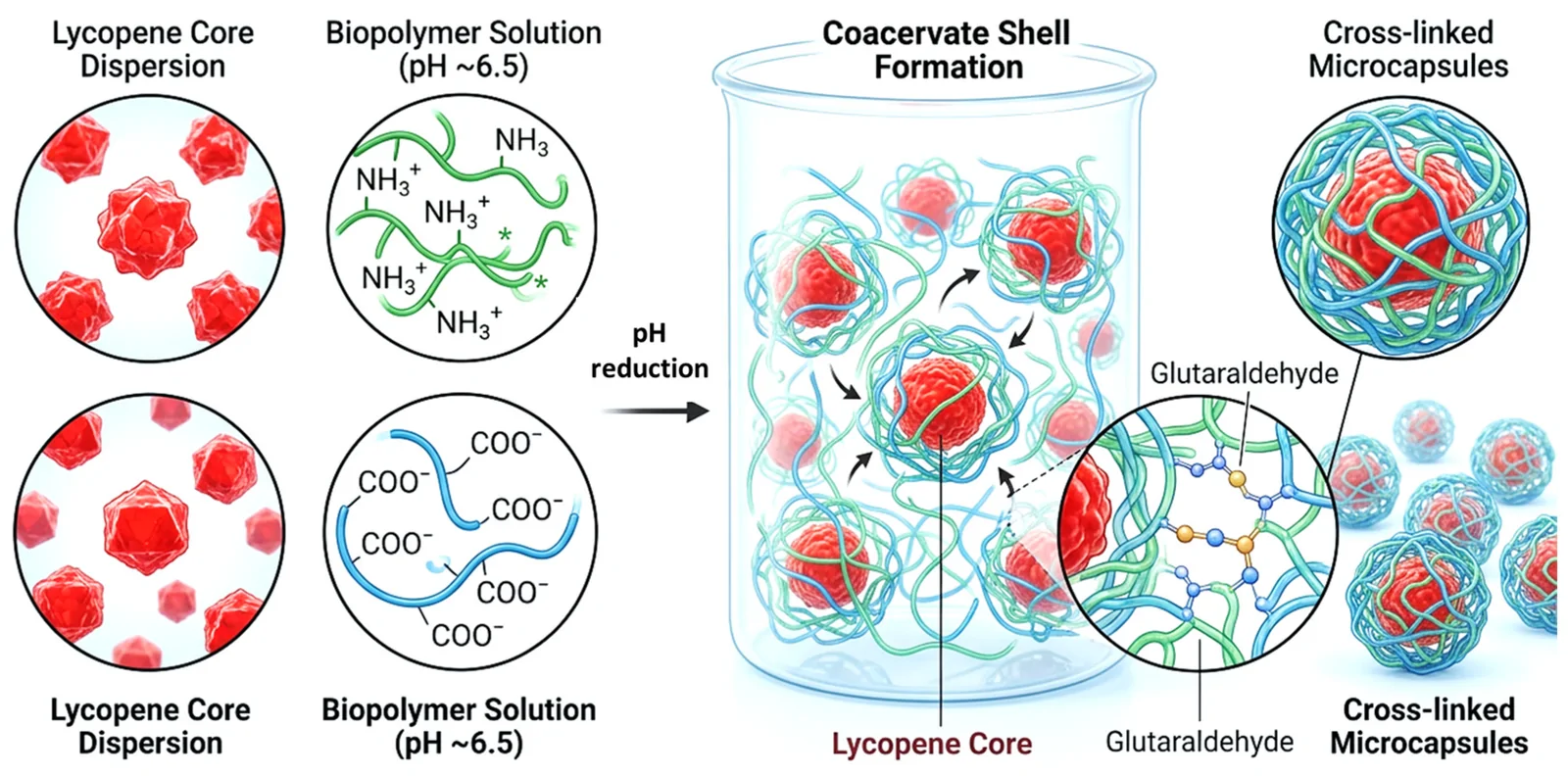
The main principles of encapsulation using coacervation.

Microencapsulation via coacervation is a technique that involves the dispersion of a bioactive compound in an aqueous polymeric solution; due to electrostatic interactions occurring between biopolymers with opposite charges in an aqueous environment [31], two phases are formed: a concentrated coacervate phase containing polymers and bioactive compounds, and another liquid phase with a lower polymer content (Figure 6).

Two methods are used, namely simple and complex coacervation. The simple method involves, in general, a water deficit or dehydration, which is often accelerated by the addition of salt or a desolvation fluid [42].

Spherical droplets minimize interfacial tension and have a uniform distribution under the microscope, exhibiting liquid-like behavior. In addition, they exhibit sponge-like or continuous local fluctuation architectures under advanced microscopy analysis. These particles can separate via a distinct interface, without exhibiting a physical lipid or solid polymer enclosure.

In the process of complex coacervation, dense, liquid-rich microscopic droplets are formed by the spontaneous liquid–liquid phase separation of biopolymers with opposite electrical charges (e.g., proteins, nucleic acids, polyelectrolytes) in an aqueous solution. These droplets provide a membraneless form of compartmentalization driven primarily by electrostatic attraction and entropy gain from counter-ion release [43].

Complex coacervation is widely used for encapsulation of bioactive compounds, leading to a higher encapsulation efficiency, particularly in combination with other methods, such as freeze-drying. For example, Neagu et al. [45] used complex coacervation in combination with freeze-drying to develop customized delivery systems for oleoresins from sea buckthorn. The wall materials consisted of whey protein isolates and casein, with and without a transglutaminase-mediated cross-linking reaction. In general, for coacervation, the oleoresins were added to the biopolymeric solutions, followed by pH adjustment for coacervation and freeze-drying. The authors suggested high encapsulation efficiencies of 94.55 ± 0.43% and 96.40 ± 0.72% for lycopene in native and cross-linked matrices, respectively. It seems that the whey protein-casein network produced by enzymatic cross-linking allowed the exposure of naturally buried hydrophobic groups, leading to more active hydrophobic patches for lycopene binding. The samples showed different lycopene concentrations of approximately 105 mg/g DW and 69 mg/g DW in the non- and cross-linked matrices, respectively. These authors reported good stability of lycopene during gastric digestion, with a release of a maximum of 9% from both matrices. However, significant differences appeared in the intestinal environment, when a controlled release of 47% and 57% of lycopene from the native and cross-linked matrices was observed.

The combined microencapsulation methods allowed obtaining spherosomes with different diameters, according to the matrix (15.23–73.41 µm in the native matrix and less than 35 µm in diameter in the case of the cross-linked matrix). The transglutaminase-mediated reaction led to a higher percentage of spherosomes (80%) with small- to medium-sized diameters, more uniform and fixed, stabilized in the biopolymer matrix. Both samples showed water activity lower than a_w_ = 0.25, indicating good biochemical stability and microbiological safety.

Gheonea (Dima) et al. [46] studied two combined microencapsulation techniques of tomato peel supercritical fluid extracts, namely emulsification combined with freeze-drying and complex coacervation combined with freeze-drying. The resulting powders were analyzed for the efficiency of microencapsulation of lycopene, the content of phytochemicals and antioxidant activity, antimicrobial activity and structural and morphological appearance. In addition, the stability to heat treatment and short-term storage was studied from the perspective of use in different applications. The spherical shape of the microparticles was highlighted, with a rounded outer surface, without concavities, with different sizes ranging from 378 nm to 3 μm and the presence of large coacervates, including small particles, suggesting a possible double encapsulation. The outer surfaces presented walls with a continuous structure, without cracks or interruptions, an attribute that is essential to ensure better protection of the microencapsulated lycopene [46]. The authors also studied the stability of the carotenoids from powders to heat treatment, in the temperature range of 110–140 °C. The results showed that lycopene degrades at a faster rate compared to other carotenoids, especially at high temperatures. Additionally, the authors demonstrated that the powder shows an inhibitory effect against α-amylase of 80%. In order to demonstrate the technological and functional added value of the microencapsulated powder, a dressing model was used, which showed increased antioxidant activity and a solid-like behavior.

However, as Nezamdoost-Sani et al. [47] explain, coacervation may have several critical drawbacks in encapsulation, including high operational costs, extreme sensitivity to environmental changes such as pH and ionic strength, and the need for chemical cross-linkers that can raise health or toxicity concerns.

### 5.2. Nanoencapsulation Methods

#### 5.2.1. Inclusion Complexes

Wang et al. [47] encapsulated lycopene using a precipitation method. In brief, the authors used β-cyclodextrin mixed with lycopene, the latter being slowly added to the warm solution to reach a molar ratio of lycopene:cyclodextrin of 1:1, followed by stirring at 50 °C and refrigeration at 4 °C. The cold precipitated complexes were recovered by vacuum filtration, followed by drying and stored in a desiccator until used. The co-precipitation method allowed reporting values for the yield and the entrapment efficiency of the inclusion complexes of 83.0% and approximately 72%, respectively.

The scanning electron micrographs of complexes showed a compact structure, different from lycopene and β-cyclodextrin in size and shape. The lycopene solubility increased linearly with the increasing concentration of β-cyclodextrin, following a classic AL type model and the inclusion complexes were formed at a stoichiometry of 1:1. These authors suggested a good storage stability of encapsulated lycopene when compared to the free lycopene. This indicated that the inclusion complexes could obviously enhance the photostability of lycopene. The stability of complexes was studied for different periods ranging from 0 to 210 min at 50 °C, whereas the photostability of the solution was investigated by irradiating under a lamp for 48 h. The antioxidant activity of the complexes increased when the concentration of the lycopene and inclusion complexes was 5 g/mL–30 g/mL, when compared with free lycopene.

The main advantages of the inclusion method are based on entrapping various molecules, preventing and protecting them from oxidation, controlling sublimation, and enhancing the sensory properties. Within these matrices, incompatible compounds are physically separated, influencing flavor stability and increasing antibacterial activity, with potential applications in the development of intelligent packaging. In general, cyclodextrin inclusion poses several drawbacks such as limited drug-loading capacity, high production costs, and potential stability issues in solution.

#### 5.2.2. Liposomal Microcapsules

The liposomal structure consists of spherical vesicles, in which the bioactive is covered by a phospholipid bilayer, due to the ability of the lipophilic tail and hydrophilic head of the amphiphilic molecules to self-assemble into bilayer structures [8]. Liposomal microcapsules were used to incorporate lipophilic lycopene into the lipid bilayer, thus improving lycopene stability from external environmental factors and protecting the bioactive from chemical and enzymatic changes [8]. Xia et al. [33] explained that liposomes are spontaneously formed when phospholipids are dispersed in aqueous solution.

**Figure 7:**
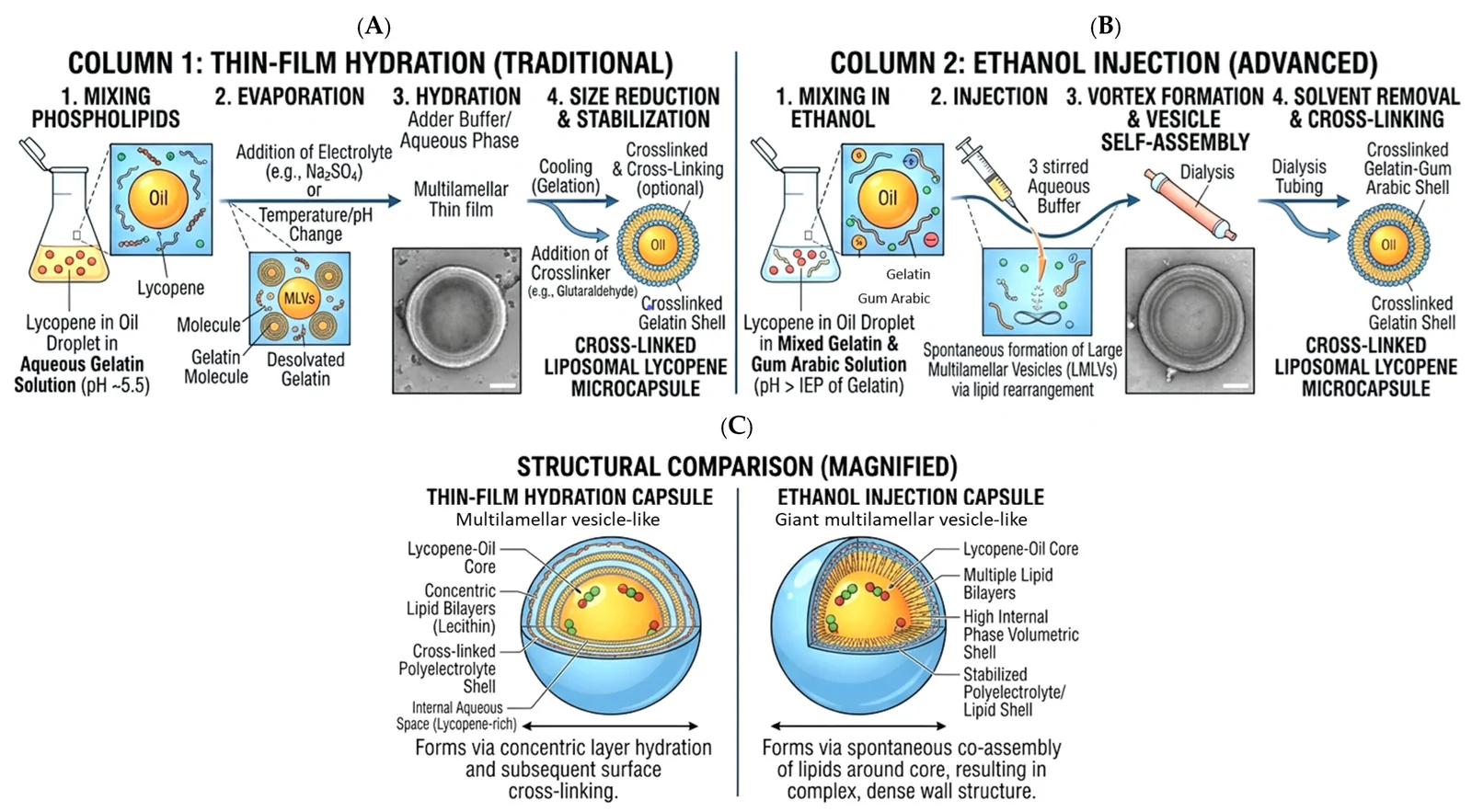
Liposomal techniques (**A**,**B**) and the main characteristics (**C**) for microencapsulation of lycopene.

Several methods are known for liposomal preparation, such as the Bangham method (which includes the dissolution of the lipophilic compounds in an organic solvent, evaporation of the solvent to deposit a dry lipid thin film and hydration of the dry film with an aqueous buffer (with or without drugs) while agitating to form multilamellar vesicles), ethanol and ether injection [48]. Different liposomal structures may be obtained, such as small unilamellar vesicles, large unilamellar vesicles, giant unilamellar vesicles, multilamellar vesicles, and multivesicular vesicles [48] (Figure 7).

Jhan & Pethe [48] used β-cyclodextrin to develop lycopene-loaded complexes. Therefore, these authors obtained double-loaded liposomes by encapsulating the lycopene-β-cyclodextrin complex in soy lecithin and cholesterol using the thin-film hydration method. The liposomal structures were characterized by an entrapment efficiency of 78.9 ± 4.8%, with a size of 255.15 ± 3 nm and zeta potential of −32.6. The formulas were stable, allowing a sustainable release up to 49% in 12 h. The saturation solubility experiments showed a higher solubility of lycopene, with a 200% increase, due to the small particle size of lycopene, which can be easily incorporated into the β-cyclodextrin inner cavity. Additionally, the lycopene-loaded formulas showed in vivo cardioprotective activity.

Xia et al. [33] investigated the ability of carotenoids to be incorporated into liposomal microparticles, using native phospholipid (egg yolk phosphatidylcholine) and nonionic surfactant Tween 80, by the thin-film evaporation method. These authors reported that the majority of particles were spherical or disk-shaped with a smooth surface, with the height of all liposomes ranging from 20 to 40 nm. However, from the carotenoids tested, it seems that lycopene incorporation led to a small increase in particle diameter, whereas other carotenoids led to a considerable increase in particle size. The technique used led to different values for encapsulation efficiency for carotenoids, ranging from the strongest to the weakest: lutein > -carotene > lycopene > canthaxanthin. The authors highlighted the susceptibility of liposomes to self-aggregation or fusion, leading to the potential leakage of entrapped compounds during storage or after administration. Thus, in order to test the stability of the liposome, they incubated the formulations at 37 °C for 5 h, evidencing the severe aggregation and fusion of lycopene-loaded liposomes, leading to the appearance of a larger number of fused particles, with a 49% increase in particle diameter. In conclusion, these authors suggested that carotenoids could modify the physical properties of the liposomal membrane in a concentration-dependent manner.

The main advantages of liposomal complexation are shown in Figure 7; in addition, it is a compatible method for encapsulating lipophilic, water-soluble, and amphiphilic materials, suitable for water activity applications, while providing highly efficient controlled delivery of bioactive compounds. The main challenges are related to the high cost, poor physical and chemical stability, wide range of particle size distribution, and complex post-treatment methods required to prevent oxidation [22].

#### 5.2.3. Niosomes

Sharma et al. [49] described a novel nano-encapsulation method for lycopene using glass wool (adsorption hydration method). The authors suggested that this method might be an attempt to preserve the lycopene activity in niosomes, using glass wool as a platform for interaction between lycopene and the wall material, which will circumvent its formulation difficulties. The resulting nano-niosomes showed particle size and size distribution in the range of 170 to 230 nm, with an average particle size of 200 ± 40 nm. Scanning electron microscopy allowed observation that the nanoparticles were spherical and morphologically similar, with an entrapment efficiency of lycopene of approximately 69%. The stability of both unloaded and lycopene-loaded nano-niosomes was monitored in terms of vesicle particle size and zeta potential during three-month storage at both 4 °C and 25 °C, showing good stability of the bioactive. The bioavailability of niosomes was evaluated by in vivo studies, using the time evolution of plasma lycopene profile after oral administration of water-suspended lycopene nanoniosomes (100 mg/kg) in rats. The relative bioavailability of lycopene nano-niosomes in rats was calculated to be 397.19% [49].

#### 5.2.4. Electrospinning

Electrospinning techniques rely on the production of nanofibers from a polymer solution, which is injected through a nozzle, where a charged polymer droplet is shaped, leading to a Taylor cone due to the electrostatic force applied [50]. The use of electrospinning leads to an increase in the nanofibers’ surface area, improving the solubility and bioavailability of nanoencapsulated bioactive compounds, compared to microencapsulated ones [50]. As explained by Belgibayeva et al. [51], in this electrohydrodynamic process, a charged polymer jet is stretched under a high electric field and solidifies into continuous fibers through rapid solvent evaporation. The method has the advantage of using moderate temperatures, thus protecting the bioactive compounds from thermal degradation, allowing encapsulation efficiency between 90.5 and 98.58%, regardless of the wall materials used [52]. One of the key factors in applying electrospinning is the high solubility and concentration of the polymer solutions, electrical conductivity, viscosity, surface tension and dielectric properties [53]. External environments such as temperature and humidity affect the morphology of the electrospun nanofibers [54]. However, Chen et al. [11] explained that the stability of the nanoparticles highly depends on the addition of synthetic surfactants like Tween-80 or natural compounds such as polysaccharides and proteins.

As a main application in the food industry, the fibers obtained by electrospinning have great potential for use in packaging, encapsulation and controlled release of bioactive compounds. In general, for fiber synthesis, different methods may be used, such as conventional methods (self-assembly, phase separation, melt blowing, wet spinning and electrospinning), but also more advanced methods such as centrifugal spinning and solution blow spinning [54].

**Figure 8:**
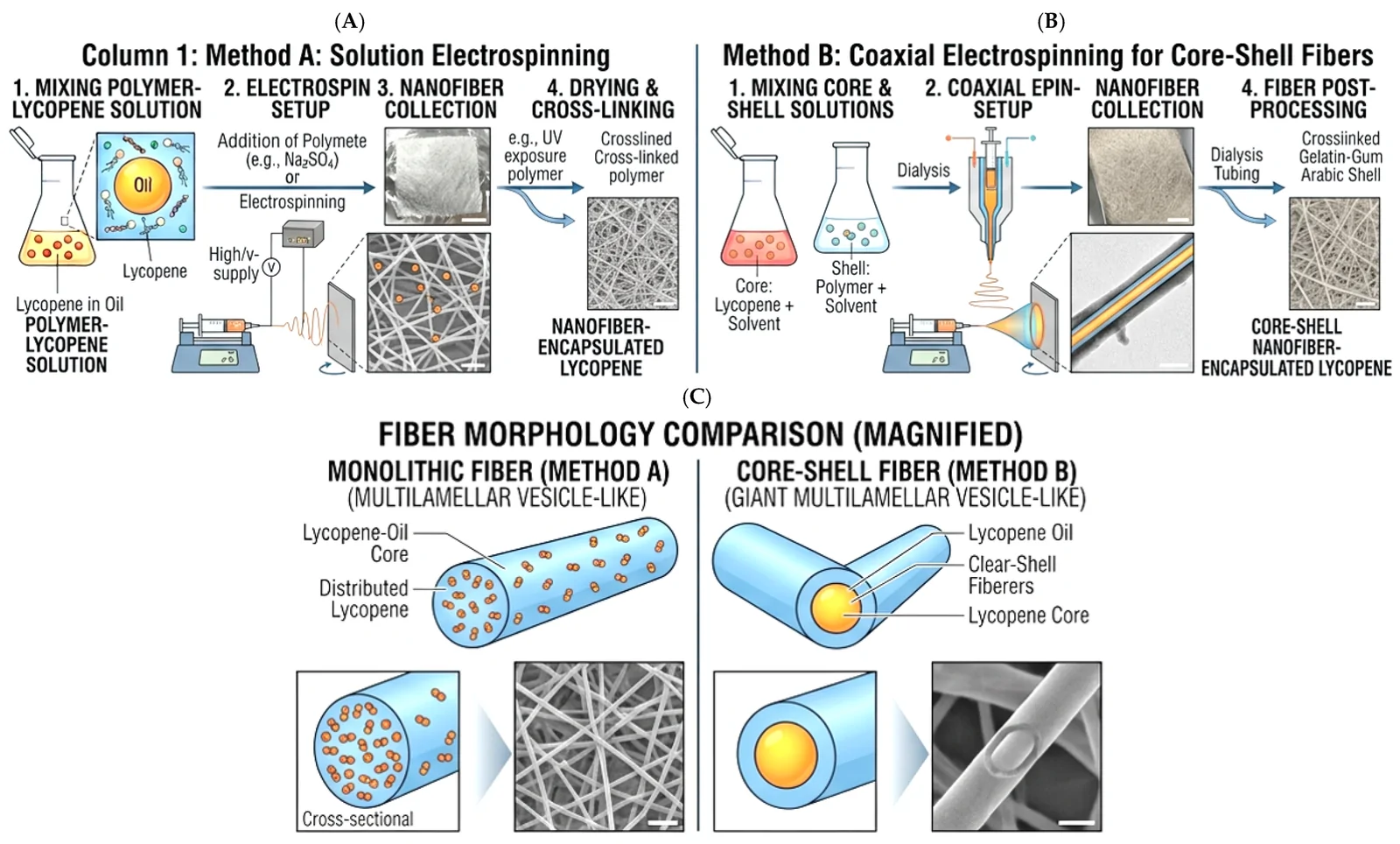
The classical and advanced methods (**A**,**B**) for electrospinning of lycopene and the structure of nanotubes (**C**).

In Figure 8, two methods are presented for the electrospinning of lycopene, together with a comparative analysis of the structure and the morphology of the nanotubes.

In Figure 8A, the solution electrospinning method is presented, consisting of using high voltage to draw a charged polymer solution into ultra-thin micro- and nanoscale fibers. Regarding the morphology, the multilamellar vesicle-like fiber (or composite multi-component/multilithic) is presented in the form of concentric or lamellar architectures, where distinct outer protective sheaths surround specialized inner lycopene core fibrils or functional layers (Figure 8).

While conventional single-fluid electrospinning produces compositionally uniform fibers, many advanced applications require precise control over internal architecture. For example, coaxial electrospinning (Figure 8B) offers new perspectives for the construction of core–shell, tubular or multilayer fibers [54]. Thus, by applying a concentric spinneret that simultaneously offers two different solutions, a spatial separation of compounds within a single filament is achieved. In Figure 8C, a shell layer can be observed, which acts as a protective and structural stabilizer, while lycopene is incorporated into the fiber interior. If a subsequent thermal treatment is applied, these composite fibers can be transformed into different architectural structures by carbonization, decomposition of phases or structural reconstruction [54].

In general, the electrospun fiber diameters typically range from 10 nm to 10 µm, with most standard polymer fibers falling in the fine 50 to 500 nm range [19]. For example, Chen et al. [11] used electrospinning to obtain lycopene-loaded nanofibers, using oil-in-water emulsions stabilized by whey protein isolate and a polysaccharide from *Tricholoma lobayense* Heim-3 (TLH). These authors suggested a maximum encapsulation efficiency of 98.75%, with an average particle size of 800 nm. The nanofibers were smooth, continuous, uniform, and bead-free, with an average diameter of approximately 225 nm. The stability of lycopene was tested during storage under different conditions, such as in the dark at −20 °C 4 °C and 25 °C. The lycopene showed a relatively high retention rate under dark conditions at −20 °C, due to its well-known sensitivity to light exposure and temperature.

The electrostatic interactions and hydrogen bonding between whey proteins and TLH-3 resulted in the formation of a tight and heavy protective layer, allowing for a cumulative release rate during in vitro digestion of approximately 27% in gastric digestion after 6 h and approximately 89% after 7 h of intestinal digestion. The authors compared the bioaccessibility of free and nanoencapsulated lycopene and suggested a 3.8-fold higher bioaccessibility of lycopene encapsulated in nanofibers (69.13% compared with 18.25%).

It is well known that in its free form, lycopene is a hydrophobic compound with poor absorption. The authors demonstrated that nanofibers significantly improved the cellular uptake efficiency of lycopene during transmembrane transport across Caco-2 cell monolayers, with efficient absorption and enhanced intracellular antioxidant activity. Yildiz et al. [55] reported the encapsulation and stabilization of lycopene by electrospinning, in the presence or absence of gelatin. The authors suggested remarkable antioxidant activity and increased thermal and UV light exposure stability of lycopene-loaded fibers. In addition, the hydrophilic nature of gelatin improved the solubility of the fibers in water, thus providing a rapid potential for oral delivery of encapsulated lycopene in fibers.

Horuz et al. [54] used electrospinning for encapsulation of lycopene into gelatin nanofibers with bead-free, smooth and homogeneous distribution of particles. These authors reported encapsulation efficiency ranging from 90.5% to 96%, as a function of gelatin concentration (5, 10 and 20%, respectively). In its encapsulated form, the lycopene showed higher stability at 4 °C in the dark when compared to 25 °C with light exposure. The higher stability was reported when stored at −20 °C, with almost no degradation of lycopene. The antioxidant activity changes in free- and lycopene-loaded nanofibers were significantly different, with a decrease of 35% and less than 27%, respectively, when stored for 14 days at different temperatures. The highest retention of lycopene antioxidant activity was found for the nanofibers stored at −20 °C. The solubility increased from 0.89% for free lycopene to 99% for nanofibers, due to the high solubility of gelatin in water, concomitant with the reduced size of the lycopene molecules to nanometric sizes. The in vitro anticancer study was performed; the results suggest that the lycopene-loaded fibers play a key role in killing the HeLa and MCF cells.

## 6. Key Factors in Encapsulation

Several methods are used for encapsulation of sensitive bioactive compounds, including lycopene. However, when choosing the parameters for encapsulation, the following key factors should be considered within the strategy to improve their functional characteristics, such as stability, solubility and systemic bioavailability [56]. These key factors are essential when comparing the efficiency of the used methods and are based mainly on the encapsulation efficiency, storage and processing stability, and release rate of lycopene in the gastrointestinal tract, thus impacting its bioaccessibility and leading to the enhanced potential for improved systemic bioavailability.

*Calculation of lycopene content* in extract or powder should use Equation (1):

(1) $$\mathrm{Lycopene}\;\mathrm{content}\;\left(\mathrm{\mu g}/g\right)=\left(\left(\frac{A_{503}}{\varepsilon }\times b\right)\times M_{w}\times \frac{V}{M}\right)\times 10^{3}$$

where

A_503_ = absorbance of the solvent phase at 503 nm;
ɛ = molar extinction coefficient (L/mol·cm) in the appropriate solvent;
b = cell optical path (cm);
MW = lycopene molecular weight = 536.9 g/mol;
V = volume of solvent (mL);
M = sample mass (g).

*Encapsulation efficiency* and *loading capacity* refer to the effective amount of bioactive entrapped into the carrier.

*Encapsulation efficiency* (*EE*) and *loading capacity* (*LC*) may be calculated according to Equations (2) and (3).

(2) $$\mathrm{EE}\;\left(\%\right)=\frac{\mathrm{Total}\;\mathrm{lycopene}-\mathrm{Surface}\;\mathrm{lycopene}}{\mathrm{Total}\;\mathrm{lycopene}}\times 100$$

(3) $$\mathrm{LC}\;\left(\%\right)=\frac{\mathrm{Total}\;\mathrm{lycopene}-\mathrm{Surface}\;\mathrm{Lycopene}}{\;\mathrm{Weight}\;\mathrm{of}\;\mathrm{microparticles}}$$

In general, for total and free lycopene content, 10 g of microencapsulated powders may be dissolved in different solvents (such as hexane, acetone, dimethyl sulfoxide, ethyl acetate, etc.). For the total lycopene content, the samples should be subjected to ultrasonication for 40 min at 100 kHz to break microparticles, whereas for the free (surface, unbounded) lycopene, it is recommended to vortex gently for 2 min. The lycopene content is calculated using Equation (1).

*Particle yield* as an additional parameter was assessed to compare the spray-dried encapsulated powder mass ratio to total solid mass in the feed before spray drying [40] (Equation (4)).

(4) $$\mathrm{Particles}\;\mathrm{yield}\;\left(\%\right)=\frac{\mathrm{Total}\;\mathrm{weight}\;\mathrm{of}\;\mathrm{obtained}\;\mathrm{particles}}{\begin{array}{c} \mathrm{Total}\;\mathrm{solids}\;\mathrm{weight}\;\mathrm{in}\;\mathrm{the}\;\mathrm{feed} \end{array}}\times 100$$

*Lycopene retention*, describing the bioactive stability during storage, may be calculated according to Equation (5).

(5) $$\mathrm{Lycopene}\;\mathrm{retention}\;\left(\%\right)=\frac{\mathrm{Lycopene}\;\mathrm{content}\;\mathrm{after}\;\mathrm{storage}}{\begin{array}{c} \mathrm{Lycopene}\;\mathrm{content}\;\mathrm{before}\;\mathrm{storage} \end{array}}\times 100$$

- *The stability of encapsulated bioactive* compounds under different storage and processing conditions is essential for their further use in different applications. An efficient encapsulation method should ensure the maintenance of the initial physicochemical properties of the compound and not facilitate its degradation under different external factors, such as temperature, pH and light.
- *Controlled release* of the entrapped bioactive during digestion, in all four stages, such as oral, gastric, small intestine and large intestine, is considered critical for bioaccessibility and bioavailability of the compound. By selecting the encapsulation materials and the appropriate method, targeted delivery systems can be obtained for the different phases of the gastrointestinal tract. Two critical stages can be considered, namely the gastric phase, where the low pH and the presence of enzymes should protect the bioactive from degradation of the encapsulation material [57]. In vitro *release* implies a static digestion method, involving simulation of gastric juice and intestinal juice, as explained by Hussain et al. [40], using Equation (6):

(6) $$\mathrm{Lycopene}\;\mathrm{release}\;\left(\%\right)=\frac{\mathrm{Released}\;\mathrm{lycopene}}{\mathrm{Total}\;\mathrm{lycopene}}\times 100$$

Therefore, high stability of the delivery systems in gastric juice is desirable. The second critical point is the intestine, where, under the action of enzymes and pH, the hydrolysis of the encapsulation materials and controlled release of the compounds is possible, favoring digestion and absorption [57].

- *Bioaccessibility and bioavailability* of the targeted bioactive are essential factors affecting the choice of an adequate encapsulation method. Bioaccessibility refers to the amount of bioactive available for absorption in organisms compared to the amount initially ingested. Bioavailability represents the amount of compound effectively absorbed and available in the circulatory system. Moreover, the effective bioavailability represents the fraction of an administered drug or nutrient that reaches the systemic circulation and remains active enough to produce a therapeutic effect. There is a direct correlation between these two characteristics, with increasing bioaccessibility leading to improved bioavailability [58].

(7) $$\mathrm{Bioaccessibility}\;\left(\%\right)=\frac{\mathrm{Concentration}\;\mathrm{of}\;\mathrm{digested}\;\mathrm{lycopene}\;}{\begin{array}{c} \mathrm{Total}\;\mathrm{concentration}\;\mathrm{of}\;\mathrm{lycopene} \end{array}}\times 100$$

In order to calculate the absolute bioavailability (%), a comparison between the dose-corrected area under the plasma concentration-time curve (AUC) of a non-intravenous dose and an intravenous (IV) dose should be performed, based on Equation (8):

(8) $$\mathrm{Absolute}\;\mathrm{bioavailability}\;\left(\%\right)\;=\;\frac{{\mathrm{AUC}}_{\mathrm{non}-\mathrm{IV}}-{\mathrm{Lycopene}}_{\mathrm{Non}-\mathrm{IV}}}{{\mathrm{AUC}}_{\mathrm{IV}}-{\mathrm{Lycopene}}_{\mathrm{IV}}}$$

The beneficial effects of lycopene on human health depend not only on the amount ingested—which varies significantly across populations—but also on the extent to which lycopene becomes available within the body. The absorption mechanism involves multiple stages of digestion and physiological processes. In the first stage, lycopene must be released from the matrix during digestion; subsequently, due to its specific properties, it undergoes emulsification and forms mixed micelles to facilitate absorption via intestinal cells. Various forms of processing—such as extraction, heat treatment, light exposure, or encapsulation can significantly enhance or reduce bioavailability. Furthermore, physiological processes, including bile secretion, digestive enzyme activity, and lipid metabolism, play a critical role in effective micelle formation and transport into the bloodstream [59].

One of the most important key factors in the bioaccesibility and bioavailability of lycopene in humans is its structural conformation. The lycopene derived from plants predominantly exists in the all-*trans* (E) configuration, accounting for approximately 80–97% of its total content. However, food sources rich in Z-isomeric forms are superior in terms of bioavailability, because lycopene is found in human plasma and tissues in the *cis* (Z) isomeric form, in a ratio of more than 50% [60]. Moreover, lycopene is found in nature in 72 isoforms, and about 90–98% of lycopene exists mainly in the all-E lycopene form [61], while the active form in human serum is Z-lycopene. The isomerization of all-E-lycopene to Z-lycopene, which is more bioavailable, involves transition through several isomers such as 5-Z-lycopene, 13-Z-lycopene, 9-Z-lycopene and prolycopene [62], driven by temperature, light and chemical activators. To the extent possible, this paper will address the connection between the isomer type and its bioaccessibility and bioavailability.

Several comparative studies showed an 8.5-fold higher postprandial absorption of *cis* (Z) forms in orange juice when compared with all-*trans* (E) isomers in tomato juice [63]. The isomerization of lycopene to the *cis* form may occur during processing, extraction, prolonged temperature exposure, storage, light, oxygen exposure, acidity, and other environmental changes. However, even if the *cis* form has a higher bioavailability, without applying stabilization techniques, its low solubility in water and limited absorption in the gastrointestinal tract further restrict its bioavailability, posing a significant challenge for different applications [64]. Two important factors, the food matrix containing lycopene and the specific pH levels within the digestive tract, directly influence lycopene bioavailability.

Upon contact with the pH of the gastrointestinal tract, all-E (*trans*) forms isomerize into Z (*cis*) forms, thereby enhancing absorption. Several studies showed that the 5-Z isomer showed the highest absorption rate and underwent minimal conversion within the intestines of meals containing 70% lycopene primarily in Z-isomeric forms [65].

However, when considering the bioavailability of lycopene, the majority of studies in the literature rely on the standardized in vitro INFOGEST digestibility method. However, the pathway a compound follows within the human body is multifactorial, ranging from bioaccessibility in the gastrointestinal tract and intestinal absorption via epithelial cells to bioavailability in the bloodstream and clinical efficacy in treating or preventing specific conditions. The use of the INFOGEST method—which mimics human gastric acids and gut enzymes—has limitations and fails to provide a true picture of the mechanisms occurring within the body. For this reason, in vivo tests are relevant, yet they have significant limitations, such as studies on humans being expensive, time-consuming, and subject to strict ethical regulations. However, in vivo bioassays using animal models, such as rats and mice, are widely adopted for assessing the relative bioavailability of different compounds. Compared to animal studies, in vitro methods are simple, faster, and cheaper, allowing an estimation of bioavailability. However, these in vitro tests cannot be conclusively used to measure and predict compound effective bioavailability until a robust correlation is established between in vivo relative bioavailability and in vitro bioaccessibility (the so-called in vivo-in vitro correlation) [66]. In the case of lycopene, even though the in vitro tests successfully predict bioaccessibility, the in vitro-in vivo correlation may be weaker due to the extreme chemical lability and its hydrophobic nature, making it difficult to replicate accurately [67].

Moreover, the size of the nanoparticles offers superior stability, controlled release, and enhanced bioavailability compared to microencapsulation. Li et al. [60] suggested that the nanometric dimensions provide higher surface area-to-volume ratios, higher dispersibility in aqueous systems, and efficient cellular uptake through transcellular and paracellular pathways. Zeta potential significantly influences the bioavailability of nanoparticles, since the surface charge dictates the way in which the nanocarriers interact with complex biological environments, such as gastric juice, intestinal mucosa, cell membranes and systemic circulation [68]. As lycopene is neutral, it is expected to act as an intestinal mucosa-penetrating particle. At this level, lycopene encounters minimal electrostatic interaction, allowing it to diffuse rapidly through the membranes to reach the underlying epithelial cells [66]. Lycopene enters cells primarily through passive diffusion or with the help of specialized membrane transport proteins like Scavenger Receptor class B type 1 and CB36 [66]. Regarding the bloodstream, lycopene inhibits non-specific protein binding, creating a “camouflage” effect, thereby prolonging circulation time and enhancing delivery potential to target tissues [58].

Another major concern of nanoparticles is related to regulatory issues regarding the toxicity of the nanocarriers; however, bioeconomy and circular economy approaches allow the obtainment of sustainable, food-grade nanocarriers and renewable materials. Regardless of the carrier and size, some important drawbacks of nanoencapsulation technology are related to high-energy input, limited loading capacity (~5–10%), low gastrointestinal stability, phospholipid oxidation, sterilization challenges, pH sensitivity, denaturation risk, allergenicity concerns, etc.

## 7. Conclusions

Different encapsulation techniques have been studied due to the need to protect and preserve biocompounds against oxidation, isomerization, and degradation during storage of healthy foods for an extended period. Lycopene, a natural compound that can be extracted from various plant materials, is unstable when exposed to different external environmental conditions and shows low bioaccessibility and bioavailability in the gastrointestinal tract. The encapsulation efficiency and stability of lycopene in different matrices highly depend on the encapsulation method used, which has high potential for bioaccessibility and bioavailability in the human body, concomitant with the well-known health benefits. Depending on the field of application, microencapsulation, nanoencapsulation techniques, or combinations of these methods can be used. A key issue also refers to the wall material selection, in terms of the ability to bind and protect lycopene, while allowing a controlled release in the gastrointestinal tract. Some key issues in bioavailability and mechanism of absorption of lycopene isomers were discussed. A number of studies and encapsulation methods have been described to stabilize lycopene, briefly presented in this chapter. However, a number of aspects need to be studied in the future, especially those relating to the toxicity and other possible side effects of the derived particles, especially in the case of nanoencapsulation. From the data presented in the literature, it seems that nanoencapsulation techniques are undoubtedly gaining ground in different fields, such as food, pharmaceutical, cosmeceutical and food supplements, with the clear advantage of superior bioavailability. However, the scaling up of these methods to an industrial scale must comply with strict regulations, especially regarding the physicochemical characterization and risk assessment for each material used individually or in combination. Moreover, the lack of standardized methods for characterization of delivery systems, standardized gastrointestinal/bioaccessibility assessment, safety and regulatory evaluation and validation in real food matrices and human clinical studies presents significant issues to be considered. The use of these encapsulated structures in more complex food systems or in real systems and clinical trials is limited. Thus, it is necessary to complete the already available data with information regarding the lycopene bioaccessibility, bioavailability and toxic or secondary effects in real systems. Another important aspect concerns the use of environmentally friendly, biodegradable materials, which may offer added economic value, such as the by-products resulting from the food industry.

## Figures and Tables

**Table 1:** Selected sources, methods and parameters for lycopene extraction.

Food By-Products	Extraction Methods	Variables	Lycopene Recovery and Optimal Conditions	References
Tomato peels from industrial waste	Solid–liquid extraction (mixture of solvents (hexane, acetone, and ethanol)	Solvent/sample ratio, number of extractions, temperature, particle size, and time	1.98 mg/100 g lycopene from the waste	[17]
Industrial tomato skins	Supercritical CO_2_ extraction	Temperature (60–80 °C), pressure (250 and 300 bar), solvent flow rate (0.792 and 1.35 kg/h), and the role of lipids, represented by tomato seeds, in the extraction of lycopene.	80% recovery of lycopene at 300 bar and 80 °C, using about 130 g of CO_2_/g matrix	[18]
Matrix composed of tomato seeds oil tomato peels and seeds	Supercritical CO_2_ extraction	The effect of temperature (70–90 °C), pressure (20–40 MPa), and CO_2_ flow rate (2–4 mL/min) on lycopene recovery from tomato peel in the presence of tomato seed oil as a co-solvent.	The optimum conditions were 90 °C, a pressure of 40 MPa, a particle size of 1.05 ± 0.10 mm, and a ratio of tomato peel to seed of 37/63. A recovery index for lycopene of 56% was obtained.	[19]
Industrial tomato waste (peel and seed)	Solvent-enzymes extraction.	Selected solvent-enzyme system optimized by Response Surface Methodology to maximize the lycopene concentration. (acetone, ethylacetate, ethanol and mixtures) and PectinexUltra SP-L, Celluclast, Viscozyme L.	Oleoresin with a concentration of 11.5 mg lycopene/g was obtained at the optimum conditions.	[20]
Tomato peels	Single-step (solid–liquid acetone extraction) and cascade approaches integrating novel technologies such as Pulsed Electric Field (PFE) and Ultrasound assisted (US) for lycopene extraction	A two-factor face-centered central composite design was used to investigate the effects of extraction time (15–60 min) and ultrasonic power (100–400 W), maintaining a temperature of 25 °C, using acetone as the extraction solvent, a solid-to-solvent ratio of 1:40 g/mL, and an agitation speed of 160 rpm. The PEF treatments were conducted with consistent field strength (5 kV/cm) and energy input (5 kJ/kg), with a fixed pulse repetition frequency (10 Hz) and pulse width (20 μs). The initial sample temperature was set at 20 ± 2	Extracts contained lutein, all-trans-lycopene, lycopene isomers, and β-carotene. PEF treatment allowed a relative increase, with a 49% lycopene recovery compared to the respective SLE, reaching a concentration of 5.42 mg/g DW in the extract.	[21]

## Data Availability

No new data were created or analyzed in this study. Data sharing is not applicable to this article.

## References

[1] Chaachouay N., Zidane L. (2024). Plant-derived natural products: A source for drug discovery and development. Drugs Drug Candidates.

[2] Masyita A., Hardinasinta G., Astuti A.D., Firdayani F., Mayasari D., Hori A., Nisha I.N.A., Nainu F., Kuraishi T. (2025). Natural pigments: Innovative extraction technologies and their potential application in health and food industries. Front. Pharmacol..

[3] Labat-Robert J., Robert L. (2014). Longevity and aging. Role of free radicals and xanthine oxidase. A review. Pathol. Biol..

[4] Lei Y., Liu H., Xiang Q., Liu Y., Wu D., Zhang J., Lin Y. (2026). Dietary lycopene mitigates reproductive impairment in heat-stressed Rongchang boars: Roles of antioxidant, anti-inflammatory and Nrf2 pathway. Antioxidants.

[5] Benabdelmoumene D., Dahmouni S., Mediani A., Bengharbi Z., Qadi W.S.M., Baharum S.N., Wong P.L., Abas F. (2026). Advancing lycopene research: Health benefits, technological applications, and emerging strategies for optimization. J. Funct. Foods.

[6] Sen L., Chen G., Zhang C., Wu M., Wu S., Liu Q. (2014). Research progress of natural antioxidants in foods for the treatment of diseases. Food Sci. Hum. Wellness.

[7] (2023). Market.us Report. https://market.us/report/carotenoids-market/.

[8] Gul K., Choudhury F.B., Roques-Carmes T. (2026). Recent developments in encapsulation technologies for stabilizing natural pigments in food systems: Mechanisms, selection strategies, and industrial feasibility. Food Biosci..

[9] Caseiro M., Ascenso A., Costa A., Creagh-Flynn J., Johnson M., Simões S. (2020). Lycopene in human health. Food Sci. Technol..

[10] Woodall A.A., Lee S.W.M., Weesie R.J., Jackson M.J., Britton G. (1997). Oxidation ofcarotenoids by free radicals: Relationship between structure and reactivity. Biochim. Biophys. Acta.

[11] Chen L., Xiang M., Wu F., Jiang Y., Wu Q., Zhang W., Guo W., Cai B., Liang L., Li S. (2023). Encapsulation of lycopene into electrospun nanofibers from whey protein isolate-Tricholoma lobayense polysaccharide complex stabilized emulsions: Structural characterization, storage stability, in vitro release, and cellular evaluation. Int. J. Biol. Macromol..

[12] Aredo V., Passalacqua E.S., Pratavieira S., Oliveira A.D. (2019). Formation of lycopene loaded hydrolysed collagen particles by supercritical impregnation. LWT—Food Sci. Technol..

[13] Anu Bhushani J., Anandharamakrishnan C. (2014). Electrospinning and electrospraying techniques: Potential food based applications. Trends Food Sci. Technol..

[14] FAO (2021). The State of Food and Agriculture.

[15] FAO, IFAD, UNICEF, WFP, WHO (2022). The State of Food Security and Nutrition in the World 2022. Repurposing Food and Agricultural Policies to Make Healthy Diets More Affordable.

[16] Carvalho G.C., Sábio R.M., Chorilli M. (2021). An Overview of Properties and Analytical Methods for Lycopene in Organic Nanocarriers. Crit. Rev. Anal. Chem..

[17] Kaur D., Wani A.A., Oberoi D.P.S., Sogi D.S. (2008). Effect of extraction conditions on lycopene extractions from tomato processing waste skin using response surface methodology. Food Chem..

[18] Sabio E., Lozano M., de Espinosa V.M., Mendes R.L., Pereira A.P., Palavra A.F., Coelho J.A. (2003). Lycopene and beta-carotene extraction from tomato processing waste using supercritical CO_2_. Ind. Eng. Chem. Res..

[19] Machmudaha S.Z., Winardib S., Sasakic M., Gotoa M., Kusumotod M., Hayakawa K. (2012). Lycopene extraction from tomato peel by-product containing tomato seed using supercritical carbon dioxide. J. Food Eng..

[20] Catalkaya G., Kahveci D. (2019). Optimization of enzyme assisted extraction of lycopene from industrial tomato waste. Sep. Purif. Technol..

[21] Carpentieri S., Başlı M., Siano F., Candoğan K., Ferrari G. (2026). Sustainable recovery of lycopene and cutin from tomato by-products: A comparative study of cascade extraction approaches using non-conventional technologies. Innov. Food Sci. Emerg. Technol..

[22] Sadik Ayyaril S., Shanableh A., Bhattacharjee S., Rawas-Qalaji M., Cagliani R., Ghassan Shabib A., Imran Khan M. (2023). Recent progress in micro and nano-encapsulation techniques for environmental applications: A review. Results Eng..

[23] Koo S.Y., Hwang K.T., Hwang S., Choi K.Y., Park Y.J., Choi J.H., Truong T.Q., Kim S.M. (2023). Nanoencapsulation enhances the bioavailability of fucoxanthin in microalga *Phaeodactylum tricornutum* extract. Food Chem..

[24] Sereti F., Alexandri M., Papapostolou H., Papadaki A., Kopsahelis N. (2025). Recent progress in carotenoid encapsulation: Effects on storage stability, bioaccessibility and bioavailability for advanced innovative food applications. Food Res. Int..

[25] Guo Y., Mao X., Zhang J., Sun P., Wang H., Zhang Y., Ma Y., Xu S., Lv R., Liu X. (2019). Oral delivery of lycopene-loaded microemulsion for brain-targeting: Preparation, characterization, pharmacokinetic evaluation and tissue distribution. Drug Deliv..

[26] de Freitas Santos P.D., Rubio F.T.V., da Silva M.P., Pinho L.S., Favaro-Trindade C.S. (2021). Microencapsulation of carotenoid-rich materials: A review. Food Res. Int..

[27] Both E.M., Boom R.M., Schutyser M.A.I. (2020). Particle morphology and powder properties during spray drying of maltodextrin and whey protein mixtures. Powder Technol..

[28] Vijeth S., Heggannavar G.B., Kariduraganavar M.Y., Salaün F. (2019). Encapsulating Wall Materials for Micro/Nanocapsules. Microencapsulation—Processes, Technologies and Industrial Applications.

[29] Szabo K., Teleky B.E., Ranga F., Simon E., Pop O.L., Babalau-Fuss V., Kapsalis N., Vodnar D.C. (2021). Bioaccessibility of microencapsulated carotenoids, recovered from tomato processing industrial by-products, using in vitro digestion model. LWT—Food Sci. Technol..

[30] Shu J., McClements D.J., Luo S., Ye J., Liu C. (2023). Effect of internal and external gelation on the physical properties, water distribution, and lycopene encapsulation properties of alginate-based emulsion gels. Food Hydrocoll..

[31] Souza A.L.R., Hidalgo-Chávez D.W., Pontes S.M., Gomes F.S., Cabral L.M.C., Tonon R.V. (2018). Microencapsulation by spray drying of a lycopene-rich tomato concentrate: Characterization and stability. LWT—Food Sci. Technol..

[32] Sing C.E. (2017). Development of the modern theory of polymeric complex coacervation. Adv. Colloid. Interface Sci..

[33] Xia S., Tan C., Zhang Y., Abbas S., Feng B., Zhang X., Qin F. (2015). Modulating effect of lipid bilayer–carotenoid interactions on the property of liposome encapsulation. Colloids Surf. B Biointerfaces.

[34] Ishwarya S.P., Anandharamakrishnan C., Stapley A.G.F. (2015). Spray-freeze-drying: A novel process for the drying of foods and bioproducts. Trends Food Sci. Technol..

[35] Joye I.J., McClements D.J. (2014). Biopolymer-based nanoparticles and microparticles: Fabrication, characterization, and application. Curr. Opin. Colloid. Interface Sci..

[36] Hamed I., Moradi M., Ezati P., O’Higgins L., Melendez-Martinez A.J., Matas R.F., Šimat V., McClements D.J., Jakobsen A.N., Lerfall J. (2023). Encapsulation of microalgal-based carotenoids: Recent advances in stability and food applications. Trends Food Sci. Technol..

[37] Li J., Pettinato M., Casazza A.A., Perego P. (2022). A comprehensive optimization of ultrasound-assisted extraction for lycopene recovery from tomato waste and encapsulation by spray drying. Processes.

[38] Corrêa-Filho L.C., Santos D.I., Brito L., Moldão-Martins M., Alves V.D. (2022). Storage stability and in vitro bioaccessibility of microencapsulated tomato (*Solanum lycopersicum* L.) pomace extract. Bioengineering.

[39] Jia C., Cao D., Ji S., Lin W., Zhang X., Muhoza B. (2020). Whey protein isolate conjugated with xylo-oligosaccharides via maillard reaction: Characterization, antioxidant capacity, and application for lycopene microencapsulation. LWT—Food Sci. Technol..

[40] Hussain A., Hussain M., Ashraf W., Ali K., Hussain A., Mon Phyo H., Khalid M.U., Hussain M., Lianfu Z. (2024). Encapsulation, optimization, and characterization of lycopene using soy protein isolate-peach gum conjugate as wall material. Food Biosci..

[41] Zhao S., McClements D.J., Liu X., Liu F. (2024). Natural pH and temperature- sensitive hydrogel-emulsion carriers for co-delivery of hydrophobic and hydrophilic bioactive ingredients in the gastrointestinal tract. Food Hydrocoll..

[42] Foo S.C., Yusoff F.M., Khong N.M.H. (2024). Storage and degradation kinetics of physicochemical and bioactive attributes in microalgal-derived fucoxanthin-rich microcapsules. J. Agric. Food Res..

[43] Aldana-Heredia H.F., Hernández-Carrión M., Gómez-Franco H.V., Narváez-Cuenca C.E., del Pilar Sánchez-Camargo A. (2024). Microwave-assisted extraction, encapsulation, and bioaccessibility of carotenoids from organic tomato industry by-product. Innov. Food Sci. Emerg. Technol..

[44] Neagu C., Mihalcea L., Enachi E., Barbu V., Borda D., Bahrim G.E., Stănciuc N. (2020). Cross-Linked Microencapsulation of CO_2_ Supercritical Extracted Oleoresins from Sea Buckthorn: Evidence of Targeted Functionality and Stability. Molecules.

[45] Nezamdoost-Sani N., Amiri S., Khaneghah A.M. (2024). The application of the coacervation technique for microencapsulation bioactive ingredients: A critical review. J. Agric. Food Res..

[46] Gheonea I., Aprodu I., Cîrciumaru A., Râpeanu G., Bahrim G.E., Stănciuc N. (2021). Microencapsulation of lycopene from tomatoes peels by complex coacervation and freeze-drying: Evidences on phytochemical profile, stability and food applications. J. Food Eng..

[47] Wang H., Wang S., Zhu H., Wang S., Xing J. (2019). Inclusion Complexes of Lycopene and β-Cyclodextrin: Preparation, Characterization, Stability and Antioxidant Activity. Antioxidants.

[48] Jhan S., Pethe A.M. (2019). Double-loaded liposomes encapsulating lycopene β-cyclodextrin complexes: Preparation, optimization, and evaluation. J. Liposome Res..

[49] Sharma P.K., Saxena P., Jaswanth A., Chalamaiah M., Tekade K.R., Balasubramaniam A. (2016). Novel Encapsulation of Lycopene in Niosomes and Assessment of its Anticancer Activity. J. Bioequiv. Availab..

[50] Mapelli-Brahm P., Margier M., Desmarchelier C., Halimi C., Nowicki M., Borel P., Reboul E. (2019). Comparison of the bioavailability and intestinal absorption sites of phytoene, phytofluene, lycopene and β-carotene. Food Chem..

[51] Okutan N., Terzi P., Altay F. (2014). Affecting parameters on electrospinning process and characterization of electrospun gelatin nanofibers. Food Hydrocoll..

[52] Belgibayeva A., Zhumabekova A., Rakhmetova A., Mukanova A., Kong L., Bakenov Z., Kim S.S., Nurpeissova A. (2026). Review on hierarchical fibers from plastic waste via coaxial electrospinning and thermal transformation: A methodological framework for energy storage applications. Chem. Eng. J. Adv..

[53] Vigilato Rodrigues M.A., Romanelli M., Bertolo V., Horn M.M., Lugão A.B., Capparelli Mattoso L.H., de Guzzi Plepis A.M. (2024). Comparing solution blow spinning and electrospinning methods to produce collagen and gelatin ultrathin fibers: A review. Int. J. Biol. Macromol..

[54] Li Q., Li P., Liu Z., Zhang J., Zhang H., Yu W., Hu X. (2024). Fabricating micro/nanostructured separators and electrode materials by coaxial electrospinning for lithium-ion batteries: From fundamentals to applications. Wuli Huaxue Xuebao/Acta Phys.—Chim. Sin..

[55] Horuz T.I., Belibağli K.B. (2018). Nanoencapsulation by electrospinning to improve stability and water solubility of carotenoids extracted from tomato peels. Food Chem..

[56] Yildiz Z.I., Topuz F., Kilic M.E., Durgun E., Uyar T. (2023). Encapsulation of antioxidant beta-carotene by cyclodextrin complex electrospun nanofibers: Solubilization and stabilization of beta-carotene by cyclodextrins. Food Chem..

[57] Saifullah M., Shishir M.R.I., Ferdowsi R., Tanver Rahman M.R., Van Vuong Q. (2019). Micro and nano encapsulation, retention and controlled release of flavor and aroma compounds: A critical review. Trends Food Sci. Technol..

[58] Bao C., Jiang P., Chai J., Jiang Y., Li D., Bao W., Liu B., Liu B., Norde W., Li Y. (2019). The delivery of sensitive food bioactive ingredients: Absorption mechanisms, influencing factors, encapsulation techniques and evaluation models. Food Res. Int..

[59] Joshi S., Dobhal A., Kaur R., Rawat M., Bhati D., Wilson I. (2025). Enhancing lycopene stability and extraction: Sustainable techniques and role of nanotechnology. J. Food Compos. Anal..

[60] Li Z., Jiang H., Xu C., Gu L. (2023). A review: Using nanoparticles to enhance absorption and bioavailability of phenolic phytochemicals. Food Hydrocoll..

[61] Rinaldi de Alvarenga J.F., Tran C., Hurtado-Barroso S., Martinez-Huélamo M., Illan M., Lamuela-Raventos R.M. (2017). Home cooking and ingredient synergism improve lycopene isomer production in Sofrito. Food Res. Int..

[62] Cooperstone J.L., Ralston R.A., Riedl K.M., Haufe T.C., Schweiggert R.M., King S.A., Schwartz S.J. (2015). Enhanced bioavailability of lycopene when consumed as *cis*- isomers from tangerine compared to red tomato juice, a randomized, crossover clinical trial. Mol. Nutr. Food Res..

[63] XiuPing L.X., Ma CuiCui M.C., Yan XiaoJia Y.X., Liu XueBo L.X., Liu FuGuo L.F. (2019). Advances in research on bioactivity, metabolism, stability and delivery systems of lycopene. Trends Food Sci. Technol..

[64] Richelle M., Lambelet P., Rytz A., Tavazzi I., Mermoud A.F., Juhel C., Bortlik K. (2012). The proportion of lycopene isomers in human plasma is modulated by lycopene isomer profile in the meal but not by lycopene preparation. Br. J. Nutr..

[65] Juhasz A.L., Smith E., Nelson C., Thomas D.J., Bradham K. (2014). Variability associated with as in vivo-in vitro correlations when using different bioaccessibility methodologies. Environ. Sci. Technol..

[66] Arballo J., Amengual J., Erdman J.W. (2021). Lycopene: A Critical Review of Digestion, Absorption, Metabolism, and Excretion. Antioxidants.

[67] Ly P.-D., Ly K.-N., Phan H.-L., Nguyen H.H.T., Duong V.-A., Nguyen H.V. (2024). Recent advances in surface decoration of nanoparticles in drug delivery. Front. Nanotechnol..

[68] Peralta-Cuevas E., Degollado-Hernández N.Y., Martínez-Ortiz I.C., Gutierrez-Onofre A.J., Garcia-Atutxa I., Villanueva-Flores F. (2026). How do nanoparticle properties shape pharmacokinetics and pharmacodynamics? A mechanistic review. Front. Pharmacol..

